# Enteric Fever in Childhood

**Published:** 1900-11-24

**Authors:** Arthur Maude

**Affiliations:** Westerham


### Hospital Clinics and Medical Progress.

### ENTERIC FEVER IN CHILDHOOD.

By Arthur Maude (Westerham).

(*Continued from page* 116.)

In a recent outbreak I have been struck by the extraordinary effect produced on the temperature particularly in children by enemata. A small enema, whether of soap-and-water or glycerine, would an hour or two afterwards produce a sudden rise of three or four degrees without any other bad symptoms.

Constipation is rather more common than in adults, and may alternate with occasional short attacks of severe diarrhœa.

It is rare, however, that diarrhœa is extreme, and it is usually easily controlled. I wish to note that I have found the naphthol compounds more efficacious in the control of diarrhœa than opium, chalk, or other usual astringent drugs. Two or three grains of beta-naphthol in the form of a powder are easily administered in milk, and seem to cause no unpleasant symptoms.

In infants under three years extreme tympanites is sufficiently common, may exist without any diarrhœa or any great constipation, and is in no way of the unpleasant importance that it augurs in older cases.

The abdomen may be so persistently distended that the diagnosis of peritonitis seems unavoidable, and yet, in spite of a high temperature and a feeble and frequent pulse, the symptoms may be misleading. Extreme tenderness all over the abdomen is not rare, and tenderness over the spleen is far more usual than in later years. The enlargement of the spleen is more easily detected in children, if indeed it is not greater and more frequently present. Taupin discovered splenic enlargement in 109 cases out of 121, but his figures are far in excess of those of Rilliet and Barthez.

Frequent short single cough is often present, especially during the first week, and scattered râles may be heard over the whole chest. The rate of respiration is often much raised without any signs of serious pulmonary mischief, and the relation between pulse rate and respiration may be greatly altered.

A respiratory or thoracic type of disease has been described, signified by dyspnœa, quickened respiration, and cyanosis, with no physical signs further than fine râles.

Great rapidity of pulse is very common in young subjects, and even if continued for several days is not of the grave import that it would indicate after youth. I have seen a child in whom a rate of 140–160 was maintained for four or five days, who nevertheless did well. West has made this observation; whereas in adults Liebermeister found that a pulse rate of 140 was accompanied by a mortality of 50 per cent., and one of 160 by a mortality of 80 per cent.

Hæmorrhage presenting the noticeable and dangerous features seen in adults is very rare in childhood (Murchison, Tanpin, Rilliet, and Barthez). The latter writers met with only one instance of hæmorrhage in 232 cases of more than average severity. But a stool containing a little black grumous remains of blood is far from a rarity. This is due to catarrhal engorgement of the mucous membrane, not to detachment of a Peyer's patch, or rupture of a vessel.

A "meningeal" type of case is met with in children, characterised by vomiting, headache, and convulsions, followed by delirium, great prostration, and torpor. Diarrhœa and involuntary discharges of fæces are common.

A girl of nine at the end of the first week had a persistent temperature of 104° Fahr.; after sponging with ice water she vomited a small quantity of bright blood. On the tenth day the head was retracted, the respiration very slow, pulse very rapid; vomited once. On the eleventh day the child was lying quite inert: eyes deeply sunken, head retracted, pulse 160, irregular, shaky; abdomen boggy, no diarrhœa. On the twelfth day the belly was quite retracted, and remained so for several days. The pulse varied from 140 to 160 for four days, the child lying inert, quite apathetic. Tache cérébrale was easily produced on the abdomen. On the eighteenth day she had a relapse of fever, after the temperature had declined daily below normal for forty-eight hours, and a copious and prolonged eruption of spots appeared.

In infantile cases strabismus and inequality of pupils may be found, and even marked opisthotonos. From the short period of many of these attacks it is unlikely that actual meningitis does occur, but it may do so, being then the result of a mixed infection.

Subsultus tendinum is not common in children, and is when present a sign of severe toxic infection, but during the long and tedious convalescence the child will often pick its nose continually till it bleeds, and render its fingers or other parts sore by picking them. Neither subsultus nor fine muscular tumor have in children the same evil significance that they have in riper years.

Delirium is uncommon in early years, though the proportional occurrence given by French observers is far higher than my own experience warrants. Taupin noted it in 44 cases out of 118, and Rilliet in one-third of his patients. It commences rather earlier than in grown subjects, probably from the earlier high range of fever, but rarely occurs before the second week.

Aphasia, generally temporary, has often been noted in children (Dreschfeld).

Convalescence is often very tedious; children are left low and weak for long periods. Not only is the bodily power and nutrition long in re-establishment, but the mental growth is arrested to a degree and for a period which excites serious alarm in the child's family. Children seem to forget how to talk, and remain in a stupid, apathetic condition for many weeks. Sometimes this condition is accompanied by deficient excretion of urea; the amount of urine is small, and of low specific gravity, with œdema of the feet, a dry, inelastic skin, and yet no albuminuria. The temperature is, however, subnormal, which is not the case in those numerous instances of late typhoid in which the kidneys are invaded by growths of streptococci.

Tuberculosis and scrofulous manifestations often have their starting-point from an attack of enteric in young subjects by heredity so disposed.

Thrombosis of the femoral veins is far less common in children.

In young children suppuration of the cervical glands about the angle of the jaw is no rare phenomenon. Harley had seen three such cases.

Catarrh and suppuration of the middle ear are sufficiently common in adult victims of enteric, but are probably even more frequent in children. Children with adenoid growths are certain to get an increase of resulting symptoms.

Inflammation of the parotid, rare on the whole in enteric (Harley; Reynold's system) is by no means uncommon in children.

In children and young persons after convalescence an exaggerated growth of the long bones frequently occurs (Dreschfeld).

The milder degrees of peripheral neuritis are as common in children as in adults. I have in these columns before raised the question whether such neuritis is not due as much to the large doses of alcohol as to the disease, but I have no sufficient data for an answer.

The prognosis in children is far more favourable than the average of all ages. The figures of mortality of the older writers cannot be accepted as proper criteria now. West lost 11 cases out of 84 at Ormond Street; Rilliet and Barthey gave the mortality as 10 per cent. in private practice, and that at the Hôpital des Enfants was at one time 25 per cent. Better feeding and nursing, especially the free use of peptonised milk, has changed all that.

Death from mere severity of the disease, so common in adults, is very rare in children. When children die from enteric they die almost invariably from perforation and general peritonitis. The greater frequency of perforation is due probably to the greater tenuity of the bowel-wall, particularly of the peritoneal coat, and also to the restlessness of the child.

"There is no other disease incidental to childhood from which recovery so often takes place in spite of the most unfavourable symptoms," says West. Quite lately a cachectic child of three, under my care, was apparently progressing towards convalescence. After a small enema she vomited, became profoundly collapsed, and for several days presented every sign of perforation and invading peritonitis except a rise of temperature. She made a good recovery nevertheless.

[The authors referred to in the text are:—Dreschfeld, art. "Allbutt's System of Medicine" (vol. i.); West, "Diseases of Infancy and Childhood;" Eustace Smith, "Disease in Children;" Harley, art. "Reynolds' System of Medicine" (vol. i.); Murchison's "Continued Fevers."]

